# Supplementary material for: Mutations in hik26 and slr1916 lead to high-light stress tolerance in Synechocystis sp. PCC6803
Source: Commun Biol. 2021 Mar 16;4:343. doi: 10.1038/s42003-021-01875-y (PMC7966805; doi:10.1038/s42003-021-01875-y)
Supplement: Supplementary file 2 — Supplementary Information [file 42003_2021_1875_MOESM2_ESM.pdf]

Supplementary Information for

Mutations in *hik26* and *slr1916* lead to high-light stress tolerance in *Synechocystis* sp. PCC6803

Katsunori Yoshikawa[1*], Kenichi Ogawa[1*], Yoshihiro Toya[1*],
Seiji Akimoto[2], Fumio Matsuda[1], Hiroshi Shimizu[1]

[1]Department of Bioinformatic Engineering, Graduate School of Information Science and Technology, Osaka University, 1-5 Yamadaoka, Suita, Osaka 565-0871, Japan
[2]Department of Chemistry, Graduate School of Science, Kobe University, 1-1 Rokkodai, Nada-ku, Kobe, Hyogo 657-8501, Japan

Correspondence: Prof. Hiroshi Shimizu, Department of Bioinformatic Engineering, Graduate School of Information Science and Technology, Osaka University, 1-5 Yamadaoka, Suita, Osaka 565-0871, Japan
E-mail: shimizu@ist.osaka-u.ac.jp

*These authors contributed equally to this work

Supplementary Methods

Construction of strains.

Single gene deletion strains of slr1916 and hik26 were constructed as described. A slr1916-deleted strains of the PCC6803 and singly-colony-isolated Tol(S1) strains, designated as 6803Δslr1916 and Tol(S1)Δslr1916, respectively, were constructed by replacing slr1916 with a chloramphenicol-resistance gene. The upstream and downstream regions of slr1916 were amplified using PCR with the primers slr1916_Up_F and slr1916_Up_R_Cm, and slr1916_Down_F_Cm and slr1916_Down_R, respectively. A KOD-plus-NEO (Toyobo, Japan) was used to perform PCR. The chloramphenicol-resistance gene was amplified from pHSG398 (Takara Bio, Japan) by using the primers Cp-F and Cp-R. The primers slr1916_Up_R_Cm and slr1916_Down_F_Cm contained overlapping regions for the amplified chloramphenicol-resistance gene. The three PCR products were connected using overlap-extension PCR (OE-PCR) using the primers slr1916_Up_F and slr1916_Down_R. The resulting PCR product was introduced into PCC6803 and Tol(S1) strains. The transformants were screened on BG11 plates containing 10-20 µg/mL of chloramphenicol, and then re-streaked on fresh BG11 plates containing chloramphenicol. Complete segregation of slr1916 by the chloramphenicol-resistance gene was confirmed by disparity in the sizes of the PCR products generated using the primers slr1916_Check_F and slr1916_Check_R, which were bound outside the amplified upstream and downstream regions of slr1916 for homologous recombination. The 6803Δslr1916/slr1916m strain was constructed as described. Mutated slr1916 (slr1916m) with its promoter region was amplified from genome DNA of the Tol(S1) strain by PCR using the primers slr1916_M_XhoI_F and slr1916_M_BamHI_R. The PCR product was cloned into pGEM-T easy, and its sequence was confirmed. The

resulting plasmid was digested by XhoI and BamHI and cloned into the corresponding site of a pSlr0168-psbA2p-me-Sm vector[1] containing a homologous region of the neutral site in slr0168 and streptomycin resistance gene. The resulting plasmid was introduced into 6803Δslr1916. The transformants were screened on BG11 plates containing 20 μg/mL streptomycin. Complete segregation was confirmed by PCR using the primers slr0168_check_F and slr0168_check_R. The hik26-deleted strains of the PCC803 and Tol(S1) strains, named PCC6803Δhik26 and Tol(S1)Δhik26, were constructed by replacing hik26 with a kanamycin-resistance gene. The upstream and downstream regions of hik26 were amplified using PCR with the primers hik26_Up_F and hik26_Up_R_Km, and hik26_Down_F_Km and hik26_Down_R, respectively. The kanamycin-resistance gene was amplified from pHSG298 (Takara Bio, Japan) by using the primers pHSG298_Km_p_F and Km_R. The three PCR products were connected by OE-PCR using the primers hik26_Up_F and hik26_Down_R. The resulting PCR product was introduced into the parent strain and the tolerant strain. The transformants were screened on BG11 plates containing 20 μg/mL kanamycin. Complete segregation of hik26 by the kanamycin-resistance gene was confirmed by PCR using the primers hik26_Check_F and hik26_Check_R. The 6803Δhik26/hik26m strain was constructed as described. Mutated hik26 (hik26m) with its promoter region was amplified from genome DNA of the Tol(S1) strain by PCR using the primers hik26_M_XhoI_F and hik26_M_BamHI_R. The PCR product was cloned into pGEM-T easy, and its sequence was confirmed. The resulting plasmid was digested by XhoI and BamHI and cloned into the corresponding site of a pNdhB-psbA2p-EtOH vector[1] containing ampicillin resistance gene and homologous regions downstream of ndhB. The resulting plasmid was introduced into 6803Δhik26. The transformants were screened on BG11 plates containing 1-5 μg/mL of ampicillin.

Complete segregation was confirmed by PCR using the primers ndhB_check_F and ndhB_check_R. The isiA overexpressed strain, named 6803/OE-isiA, was constructed as follows. isiA was amplified from genomic DNA of PCC6803 using primers isiA_NdeI_F and isiA_BamHI_R. The PCR product was cloned into pGEM-T easy, and the sequence was confirmed. The resulting plasmid was digested by NdeI and BamHI and cloned into the corresponding site of a pSlr0168-psbA2p-me-Sm plasmid containing a homologous region of the neutral site in slr0168 and a strong psbA2 promoter. The resulting plasmid was introduced into PCC6803, generating 6803/OE-isiA. The transformants were screened on BG11 plates containing 20 µg/mL streptomycin. Complete segregation was confirmed by PCR using the primers slr0168_check_F and slr0168_check_R. The primers used in this study are listed in the Supplementary Table S6.

Supplementary Figures

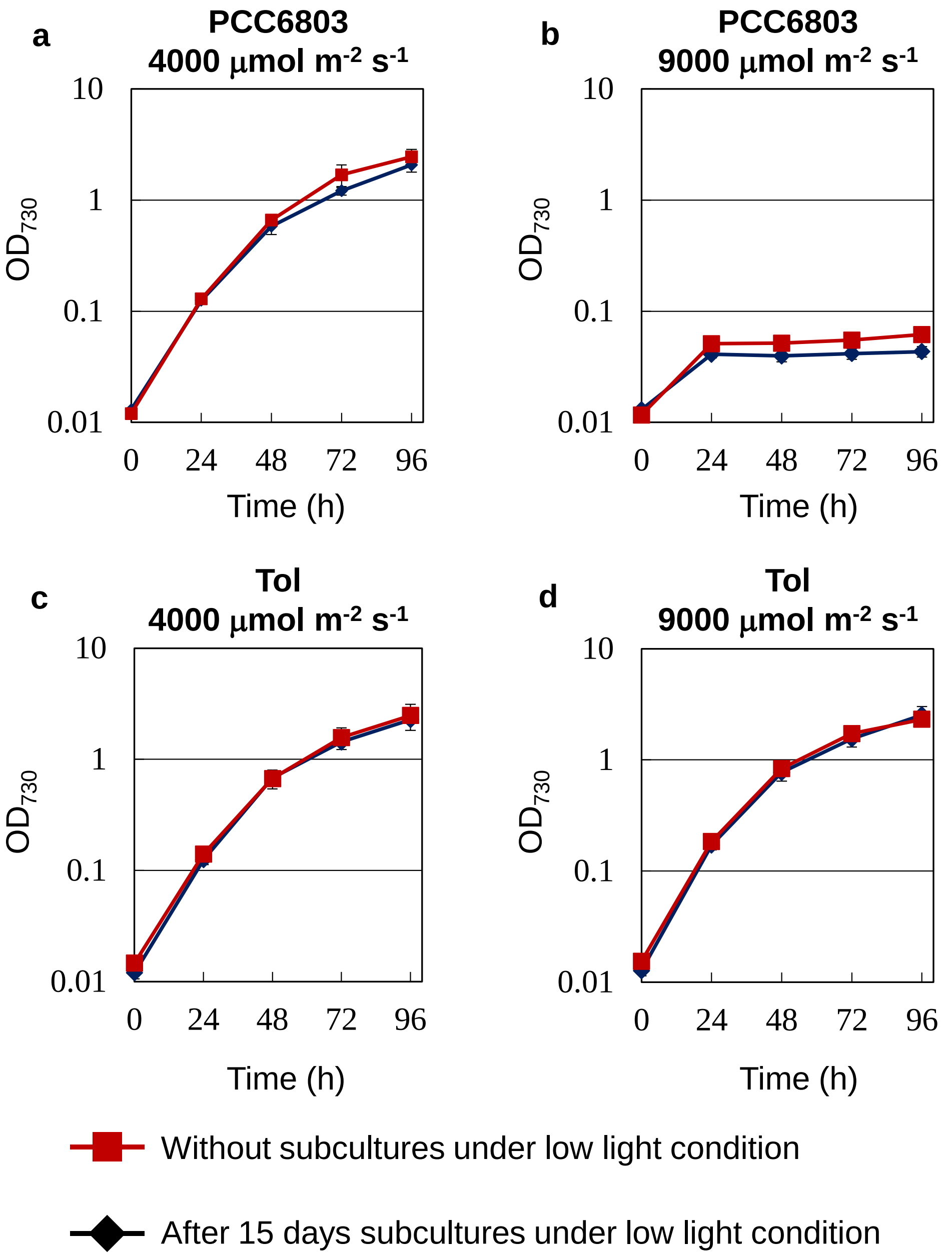

Supplementary Fig. 1 Evaluation of HL tolerance stability of the Tol strain. The PCC6803 and Tol strains were cultured under 4000 and 9000 μmol m^{-2} s^{-1} after with or without 15 days subcultures under low light condition (40 μmol m^{-2} s^{-1}). The panels of a and b, and c and d represent the PCC6803 and Tol strains, respectively. The left and right panels show growth under 4000 and 9000 μmol m^{-2} s^{-1}. Error bars indicate standard deviation of triplicate cultures.

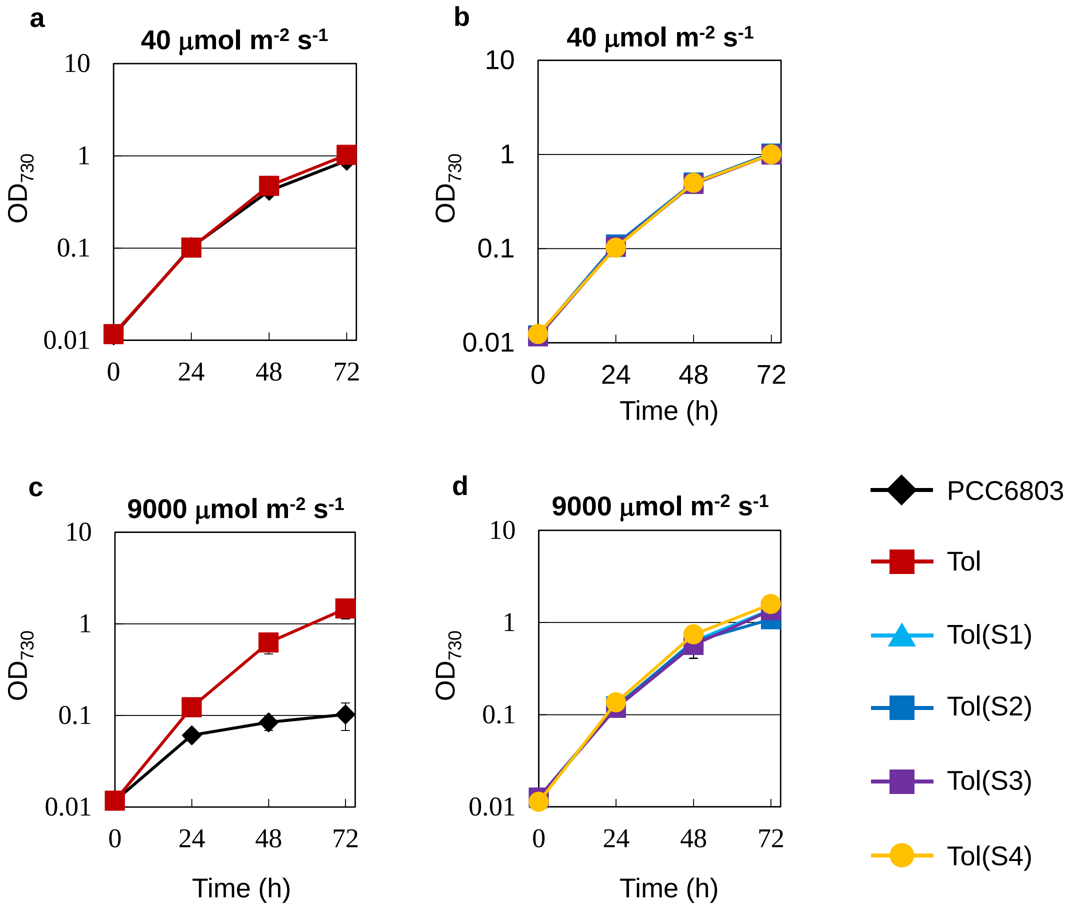

Supplementary Fig. 2 Comparison of growth curves between Tol strain and its isolated strains, Tol(S1) to Tol(S4). The graphs of a and b, and c and d represent growths under 40 and 9000 μmol m^{-2} s^{-1}, respectively. Error bars indicate standard deviation of triplicate cultures.

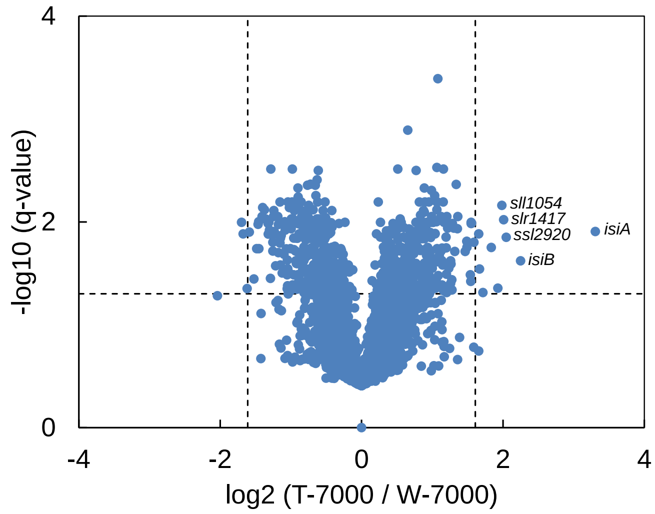

Supplementary Fig. 3 Volcano plot for finding genes whose expressions were significantly changed in terms of q-values ($\alpha < 0.05$) and the fold increase (> 5 and < 0.2).

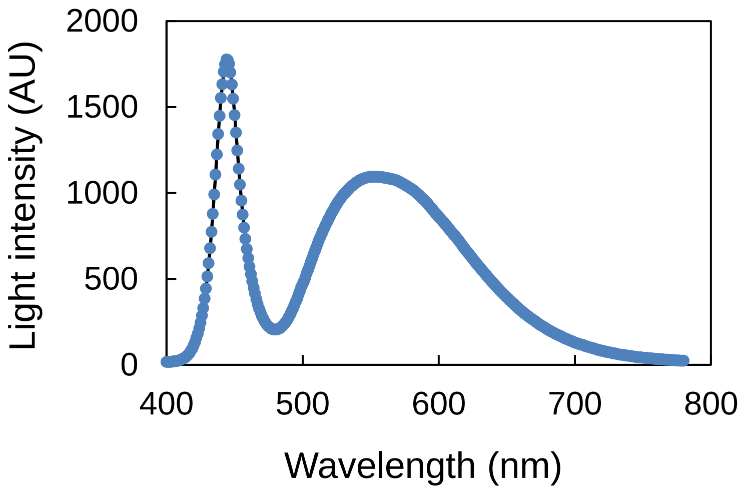

Supplementary Fig. 4 Spectral profile of the point source LED light.

Total light intensity was adjusted to 1000 μmol m^{-2} s^{-1} light. The spectral profile was measured with a light analyzer (LA-105, NK Systems, Japan).

Supplementary Tables

Supplementary Table 1 Summary of whole genome sequencing.

	PCC6803	Tol
Average read depth	432	500
Genome coverage	100%	100%
Total number of mutations*	7	9
SNPs	6	8
Transposon	1	1

* 7 of 9 mutations of the Tol strain were same as those of PCC6803.

Supplementary Table 2 Summary of SNPs and indels identified in the PCC6803 and Tol strains.

Genomic position	Reference	PCC6803	Tol	Gene	Amino acid change	Function	Comment
619877	G	G	A	*slr1916*	C53Y	Probable esterase	Found in Tol strain only
968237	T	G	G	*sll1716*	Q73L	Putative transposase	
1193085	C	A	A	*slr1855*	P167T	Unknown protein	
1277618	T	T	G	*slr1805* (*hik16*)		Two-component sensor histidine kinase	
1391505	C	T	T	*slr1250* (*pstB*)	S204L	Phosphate transport ATP-binding protein PstB homolog	
1721396	C	T	T	*sll1260* (*rpsB*)	D165N	30S ribosomal protein S2	
1763117	G	T	T	*slr1962*	C158F	Probable extracellular solute-binding protein	
2615870	C	C	A	*slr0484* (*hik26*)	T29K	Two-component sensor histidine kinase	Found in Tol strain only
3420058	G	A	A	*slr0753*	V165D	Probable transport protein	
3525512	T	C	C	*slr1187*		Unknown protein	
3525518	T	C	C	*slr1187*		Unknown protein	
3525495	A	C	-	*slr1187*		Unknown protein	
2055887		indel	indel	*sll1527*	-	Unknown protein	1727861- 1729034 Identities = 1172/1174 (99%), Gaps = 0/1174 (0%) including sll1255 (SYNGTI_1580)

Supplementary Table 3 Strains used in this study.

Strain	Relevant genotype*
PCC6803	*Synechocystis* sp. PCC 6803 Glucose-tolerant
Tol	HL tolerant strain of PCC6803 obtained by adaptive evolution experiment
Tol(S1)	Single colony No. 1 of Tol strain
Tol(S2)	Single colony No. 2 of Tol strain
Tol(S3)	Single colony No. 3 of Tol strain
Tol(S4)	Single colony No. 4 of Tol strain
6803Δhik26	*hik26*::Km^r
6803Δhik26/hik26m	*hik26*::Km^r, NS1::*hik26m*, Am^r
6803Δslr1916	*slr1916*::Cm^r
6803Δslr1916/slr1916m	*slr1916*::Cm^r, NS2::*slr1916m*, Sm^r
Tol(S1)Δhik26	Tol(S1), *hik26*::Km^r
Tol(S1)Δslr1916	Tol(S1), *slr1916*::Cmr
6803/OE-isiA	NS2::psbA2p-*isiA*, Sm^r

* NS1, neutral site downstream from *ndhB* [2]; NS2, neutral site located in *slr0168* [3]; Km^r, kanamycin resistance gene; Am^r, ampicillin resistance gene; Cm^r, chloramphenicol resistance gene; Sm^r, streptomycin resistance gene; psbA2p, promoter of *psbA2*.

Supplementary Table 4 Primers used in this study.

Primer	Sequence (from 5' to 3' end)
slr1916_Up_F	TGGTTACTGAACTGGGTTAC
slr1916_Up_R_Cm	CCAGTGATTTTTTTCTCCATAGTTCTCGCAATTGCTACG
slr1916_Down_F_Cm	AGTGGCAGGGCGGGGCGTAAATGGGGGCAATTGTTGGC
slr1916_Down_R	GCAGGGAACAGTTAAGAGC
Cp-F	ATGGAGAAAAAAATCACTGGATATACC
Cp-R	TTACGCCCCGCCCTGCCACT
slr1916_Check_F	TTCTGTCAATACAGAGGGTC
slr1916_Check_R	CACCAACTCATCGGTCACC
slr1916_M_XhoI_F	ctcgag TTGATCGGGATGGCAAAGTG
slr1916_M_BamHI_R	ggatcc GATTCCAGGGAGACCATAGC
hik26_Up_F	CCAAAAGTCCCCAATCACCG
hik26_Up_R_Km	GCGATTCAGGCCTGGTATGACGCTCCTGAAAAAAGGGGAG
hik26_Down_F_Km	GATGCTCGATGAGTTTTTCTAATTTATCCTAAAAAATAGCCCTTACC
hik26_Down_R	CAGTAAATCCTGTTGCAAAGC
pHSG298_Km_p_F	TCATACCAGGCCTGAATCGC
Km_R	TTAGAAAAACTCATCGAGCATC
hik26_Check_F	TTTGCCATTAGTCAAAGTGG
hik26_Check_R	CTAGATTCCAATAGATCAAGGG
hik26_M_XhoI_F	ctcgag TTTACAGTTGATCGGCATGG
hik26_M_BamHI_R	ggatcc ACGGGGACTAAAACTGGTGC
ndhB_check_F	GTGAAGATGGTTACGCCAGC
ndhB_check_R	TCCTGGAGCCCTAAAGTTGC
isiA_NdeI_F	catATGGGAGACCAGGGATTAATTC
isiA_BamHI_R	ggatcCTAGGTTTGCAAGGAATCAAACG
slr0168_check_F	CCCATCGTAAAATTCGTTCC
slr0168_check_R	CTGGTGTAAATTCGCAAACG

Supplementary References

1. Yoshikawa K, Hirasawa T, Shimizu H. Effect of malic enzyme on ethanol production by *Synechocystis* sp. PCC 6803. *J Biosci Bioeng* **119**, 82-84 (2015).
2. Takahashi T, Nakai N, Muramatsu M, Hihara Y. Role of multiple HLR1 sequences in the regulation of the dual promoters of the *psaAB* genes in *Synechocystis* sp. PCC 6803. *J Bacteriol* **192**, 4031-4036 (2010).
3. Angermayr SA, Paszota M, Hellingwerf KJ. Engineering a cyanobacterial cell factory for production of lactic acid. *Appl Environ Microbiol* **78**, 7098-7106 (2012).
