# Supplementary material for: Mutations in hik26 and slr1916 lead to high-light stress tolerance in Synechocystis sp. PCC6803
Source: Commun Biol. 2021 Mar 16;4:343. doi: 10.1038/s42003-021-01875-y (PMC7966805; doi:10.1038/s42003-021-01875-y)
Supplement: Supplementary file 3 — Description of Additional Supplementary Files [file 42003_2021_1875_MOESM3_ESM.pdf]

Description of Additional Supplementary Files

File Name: Supplementary Data 1

Description: Excel file for gene expression data of PCC6803 and Tol strains under 4000 and 7000 μmol m^{-2} s^{-1}

File Name: Supplementary Data 2

Description: Excel file for source data of main figures
